# Psychometric validation of the Spanish for Ecuador Family Reported Outcome Measure (FROM-16) and its application to measure impact on family members of patients with skin diseases

**DOI:** 10.1186/s41687-025-00866-5

**Published:** 2025-05-09

**Authors:** Andrea Cueva, Faraz M. Ali, Jeffrey Johns, Andrew Y. Finlay, Sam Salek

**Affiliations:** 1 Dermatology Outpatient Clinic, Hospital Carlos Andrade Marin, Quito, Ecuador; 2 InnovaDerm, Quito, Ecuador; 3 Division of Infection and Immunity, School of Medicine, Cardiff University, Cardiff, CF144XN UK; 4 School of Life & Medical Sciences, University of Hertfordshire, Hatfield, UK

**Keywords:** FROM-16, Quality of life, Family members, Partners, Dermatology, Clinical practice, Validation

## Abstract

**Background:**

The Family Reported Outcome Measure (FROM-16) is a generic tool to measure the impact of health conditions on patients’ family members and partners (FMs). This study aimed to translate, validate, evaluate, and implement FROM-16 in Ecuadorian Spanish and to assess the quality of life (QoL) of FMs of patients with skin diseases.

**Methods:**

A cross-sectional study of patients and their FMs was performed using FROM-16 and a five-point-Likert scale to evaluate patient´s skin health by the FM and physician. Construct validity, confirmatory factor analysis and item response modelling of FROM-16 were assessed.

**Results:**

195 FMs completed Ecuadorian FROM-16. Inter-item correlation was 0.40 and factor analysis confirmed the original two-factor model: Cronbach’s alpha 0.89, with factor loadings of 0.44–0.76. Mean age of patients = 41.8 ± 31.1 years and of FMs = 47.3 ± 7; diseases were classified as inflammatory (*n* = 88) or non-inflammatory (*n* = 107). The mean FROM-16 score was 12.5 ± 7 meaning “a moderate effect on QoL;” however, scores of 29.2% (57 of 195) indicated a “very large” (*n* = 47) or “extremely large” (*n* = 10) effect. Populations with the highest burden were adult children main carers, not cohabiting with their sick parents (mean FROM-16 = 17 ± 7.7, *n* = 8, *p* = 0.05 versus those cohabiting), and FM of patients with inflammatory conditions (mean = 14 ± 6.9, *n* = 88, *p* = 0.006 versus those with non-inflammatory dermatoses).

**Conclusion:**

The FROM-16 is a succinct, well-structured two-domain instrument. It can be used to identify the largely overlooked impact on FMs of dermatology patients. Understanding this impact may contribute to better holistic care, inform physicians’ decisions, and encourage further support for families.

**Supplementary Information:**

The online version contains supplementary material available at 10.1186/s41687-025-00866-5.

## Abstract (plain language summary)

This study is needed to address the lack of information on the impact of dermatoses on family members (FMs) and the absence of validated, generic quality of life (QoL) tools to assess this impact. The key issue addressed is whether the Spanish for Ecuador version of FROM-16, a family-reported outcome measure, can reliably assess the QoL impact on FMs of patients with skin diseases. This study examines the psychometric validity of FROM-16 and assesses if the translated tool maintains consistency with the original version. Results show that the Ecuadorian FROM-16 achieves a robust inter-item correlation (0.40) and a strong two-factor model with a Cronbach’s alpha of 0.89, confirming its reliability. The findings reveal that nearly one-third of FMs experience a significant decline in QoL, particularly adult children caring for parents and those FMs of patients suffering from inflammatory skin conditions. These results underscore the substantial QoL impact on FMs and suggest that clinicians consider these effects when designing holistic care approaches.

**Supplementary Information:**

The online version contains supplementary material available at 10.1186/s41687-025-00866-5.

## Introduction

Health involves families: when all in a family are physically and emotionally healthy, families thrive; but when someone is ill, then other family members and partners (FMs) are also affected [1]. When caring for someone who is ill, the main family caregiver may be most affected, experiencing depression, anxiety and feelings of hopelessness [2] with an effect on their emotional, personal, and social life; [3, 4] yet, all FMs may be affected.

A dermatology-specific measure, the Family Dermatology Life Quality Index [5] and several disease-specific tools have been developed to measure this family burden, such as for psoriasis [6, 7], atopic dermatitis [8–12], epidermolysis bullosa [13] and ichthyosis [14]. However these tools are not widely used and family burden may be overlooked [15]. The Family Reported Outcome Measure (FROM-16) [16, 17] is a generic tool with score band meaning descriptors [18] that can be used across medicine, allowing comparison between the impact of disease on the quality of life (QoL) of FMs in dermatology and other medical specialties. The objective of this study was to translate and validate the FROM-16 in Spanish in Ecuador, to assess the impact of skin disease on the FMs including partners of outpatients in dermatology and to compare FROM-16 values with previous reports.

## Methods

### Ethical aspects

Ethical permission was approved by the Ethics Committee of Hospital Carlos Andrade Marín (Acta 008-20-Abr-2023). All FMs and patients gave informed written consent to participate in this cross-sectional study.

### Cross-cultural adaptation of FROM-16 into Ecuadorian Spanish

#### FROM-16

The FROM-16 [16, 17] includes 16 items grouped into two domains: one which assesses the emotional state (items 1 to 6) and another which focusses on the impact on social and personal life (items 7 to 16). Each item is answered using a three-point Likert scale (English: not at all, a little, a lot; Spanish: nada, poco, bastante) with scores 0, 1 or 2 points, respectively. The FROM-16 also has an established range of score banding descriptors [18]: no effect (0–1), small effect (2–8), moderate effect (9–16), very large effect (17–25) and extremely large effect (26–32) on the QoL of FMs.

#### Translation, content validity and practicality

The translation of FROM-16 from the original British English to Spanish for Ecuador followed an interactive forward-backward translation process [19]. Two independent translators created two forward translations, which were peer-reviewed and reconciled among the translators and a bilingual expert in the field; the meaning and clarity of the questions, as well as issues concerning the appropriateness of questions were considered. Then, two further independent bilingual translators created two backward translations for review by the developers. Inconsistencies were discussed and resolved by the developers and the bilingual expert. After final confirmation by the developers, the two forward translations were reconciled into one final forward translation. In a pilot study, 30 FMs completed the pilot FROM-16 survey to check for any difficulties in the questions wording: no revisions were needed.

A further study was carried out in a single tertiary referral centre to demonstrate the feasibility of use of FROM-16, verifying its practical potential and internal consistency reliability.

#### Cognitive debriefing

Family members were asked to answer seven cognitive debriefing questions about the face validity of the FROM-16: Did you find the questions clear and straightforward to respond? Did you find the response options relevant to the heading of each section? Did you find the questions relevant to the aims and objectives of the study? Did you find the questions relevant to you as a family member of a person with a skin disease? Did you find any relevant questions missing? If yes, which; did you find any questions not relevant that should be excluded? If yes, which; did you find the questionnaire useful to reflect the impact of your family member’s skin disease on your quality of life?

Family members were selected by asking the accompanying relative to take part in the study. Where a patient was accompanied by more than one family member, the main carer was chosen. Family members were informed that the patient would not see their responses, in order to reduce bias of responses.

#### Selection of study participants

Adults and children with any dermatoses and their adult FMs were approached at a tertiary care centre in Quito, Ecuador. Both FMs and patients were provided with information sheets and consent forms. Patients were included if they were able to read, write and understand Spanish, and capable of giving written informed consent or assent for their FM to participate. Patients with other chronic coexisting medical conditions were excluded. The inclusion criteria for FMs were: being 18 years of age or older, able to read and understand Spanish, able to give written informed consent and complete the interview and questionnaires.

### Data collection

A single researcher (AC) asked and recorded demographic data (age and gender) of FMs and patients, also relationship to the patient, whether the FM cohabited with the patient, whether the FM was the main caregiver, the type of skin disease and the current skin health status of the patient using a five-point Likert scale ranging across very poor, poor, fair, good, and very good. A short explanation regarding the FROM-16 and its usefulness was given to the FM, and they were advised to complete the questionnaire based solely on the skin disease. The FM then completed the questionnaire and also graded the current skin health status of the patient using the five-point Likert scale.

### Statistical analysis

Anonymous research data was entered onto Google^®^ forms and downloaded into an Excel^®^ file for analysis. Data was entered in Excel and analysed using IBM SPSS Statistics version. Mean (M), standard deviation (SD), and median (Md) FROM-16 scores were calculated. To test reliability, Cronbach’s alpha (internal consistency) was calculated for each of the two FROM-16 subscales and summary scores. Spearman´s rank correlation coefficient was used to examine the relationship between variables.

All research documents and data are kept secure in the Department of Dermatology of Hospital Carlos Andrade Marin: only the key researchers have access to the data.

### Psychometric analysis

Construct validity was measured: this is defined as the degree to which the scores of a measurement instrument adequately reflect the dimensionality of the construct to be measured. Confirmatory analysis (CFA) was used to examine whether the data fit the predetermined 2-factor model. Fit parameters were used to test whether the original two-factor model was superior to alternative models. Evaluation of model fit was performed using the comparative fit index (CFI), Tucker–Lewis’s index (TLI), Root Mean Square Error of Approximation (RMSEA), and Standardised Root Mean Square Residual (SRMR) including 90% confidence intervals (CI). The CFI assesses fit relative to a null model and ranges from 0 to 1 with values of 0.90–0.95 indicating acceptable and > 0.95 good fit. The TLI adjusts for the number of model parameters and is interpreted as for CFI. The RMSEA expresses the lack of fit per degree of freedom of the model with values interpreted as follows: ≤0.05 = very good; >0.05–0.08 = good; ≥0.10 = poor fit. The SRMR is the average of the differences between the observed and predicted correlations and has a range from 0 to 1 with values < 0.08 indicating good fit. Internal consistency was determined by Cronbach’s alpha (18, 19) and was calculated using IBM SPSS Statistics version 27. Other analyses were performed in R version 4.2.2 (R Foundation for Statistical Computing); CFA using the (Lavaan package) with maximum likelihood (ML) estimator and NLMINB optimization method, parallel analysis (Psych package) and item response theory (MIRT package) using Rasch and graded response models.

## Results

For the purpose of clarity, the results will be presented in two parts: Part I, Translation, content validity, practicality and psychometric testing; and Part II, clinical application of the Ecuadorian FROM-16.

### Part I - Translation, content validity, practicality and psychometric testing

#### Forward and back translation of FROM-16 into Ecuadorian Spanish

Two forward translators (JJ, and MB in acknowledgements) translated the original FROM-16 in British English to two localised Ecuadorian versions. Small inconsistencies were found with the word ¨relative¨ versus ¨family member¨ ¨familiar¨ versus ¨miembro de mi familia,¨ and on questions 5, 6, 8, 11, 15, 16 with wording such as ¨encontrar a alguien para hablar¨ versus ¨con quien hablar¨ (q5), or ¨los gastos familiares aumentaron¨ versus ¨mis gastos familiares incrementaron¨ (q15) either holding similar meaning; these were reconciled by a third reviewer AC along with JJ and MB reaching two provisional forward translations; no serious discrepancies were found, no third translator was involved in the process. Then ACG and PC independently performed backward translations to English. FA reviewed the translation and minor British/American English language differences were found in questions 2, 5, 6, 8, 9, 15, such as ¨taking care of my family member is hard¨ versus ¨ taking care of my family member is difficult, ¨ or ¨my food habits¨ versus ¨my eating habits¨ (q9), or ¨my sexual life¨ versus ¨my sex life¨ (q12). AC and FA met to discuss the inconsistencies, but as they were minor and the meaning of the questions prevailed, AC and FA reconciled the two translations into one final forward translation in localized Ecuadorian Spanish. Then FROM-16 was implemented in 30 family members and patients in the Dermatology department of HCAM in Quito; the clarity of questions and easiness to fill out the questionnaire were assessed. No revisions were needed. After this pilot study, the main study was carried out.

#### Cognitive debriefing

Fifty-four FMs responded to the cognitive debriefing procedure: 51 stated they found the questionnaire useful to reflect their QoL. Suggestions were made for extra questions; some respondents found question 12 ¨my sex life is affected¨ inappropriate and mentioned they thought it should not be included; others mentioned that the state of mind of the patient has an influence on how questions are answered, and that the questionnaire gives insight into the current impact, but does not reflect the overall impact such as how worried one could be in the initial stages of disease, moreover the burden of disease might be different when a disease is chronic or acute. One mother with epidermolysis bullosa, whose daughter had the same diagnosis, worried about the bullying her daughter may suffer in school. Furthermore, we noticed how uninformed or confused FMs could be; for instance, one mother regarded her daughter’s diagnosis as melanoma whereas the diagnosis was melanonychia and several others were unaware of the diagnosis of their FM.

#### Psychometric analysis

Correlation between items generally was low (< 0.5) with only 7 interitem correlations between 0.5 and 0.6 and mean inter-item correlation 0.40 (Table 1). Inter-item correlation values between 0.15 and 0.50 represent a good result whereas values lower than 0.15 mean that items are not correlated well and values higher than 0.50 mean that items are correlated to a great extent and the items may be repetitive in measuring the intended construct [20].

**Table 1:**
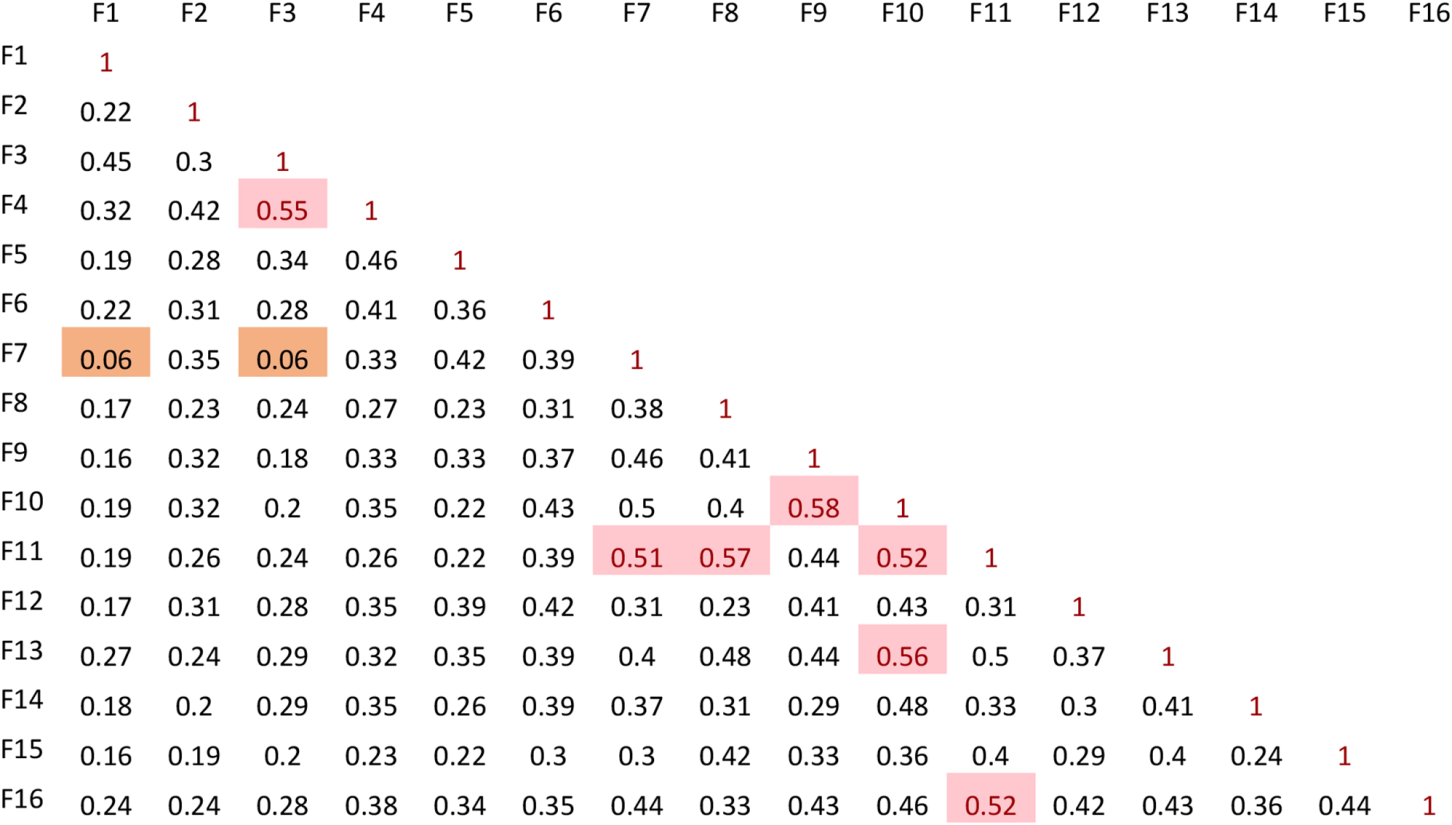
Inter-item correlation plot of the 16 items of FROM-16. Pink indicates items with correlation > 0.5 and orange items with correlation < 0.15

#### Structural validity

**Fig. 1:**
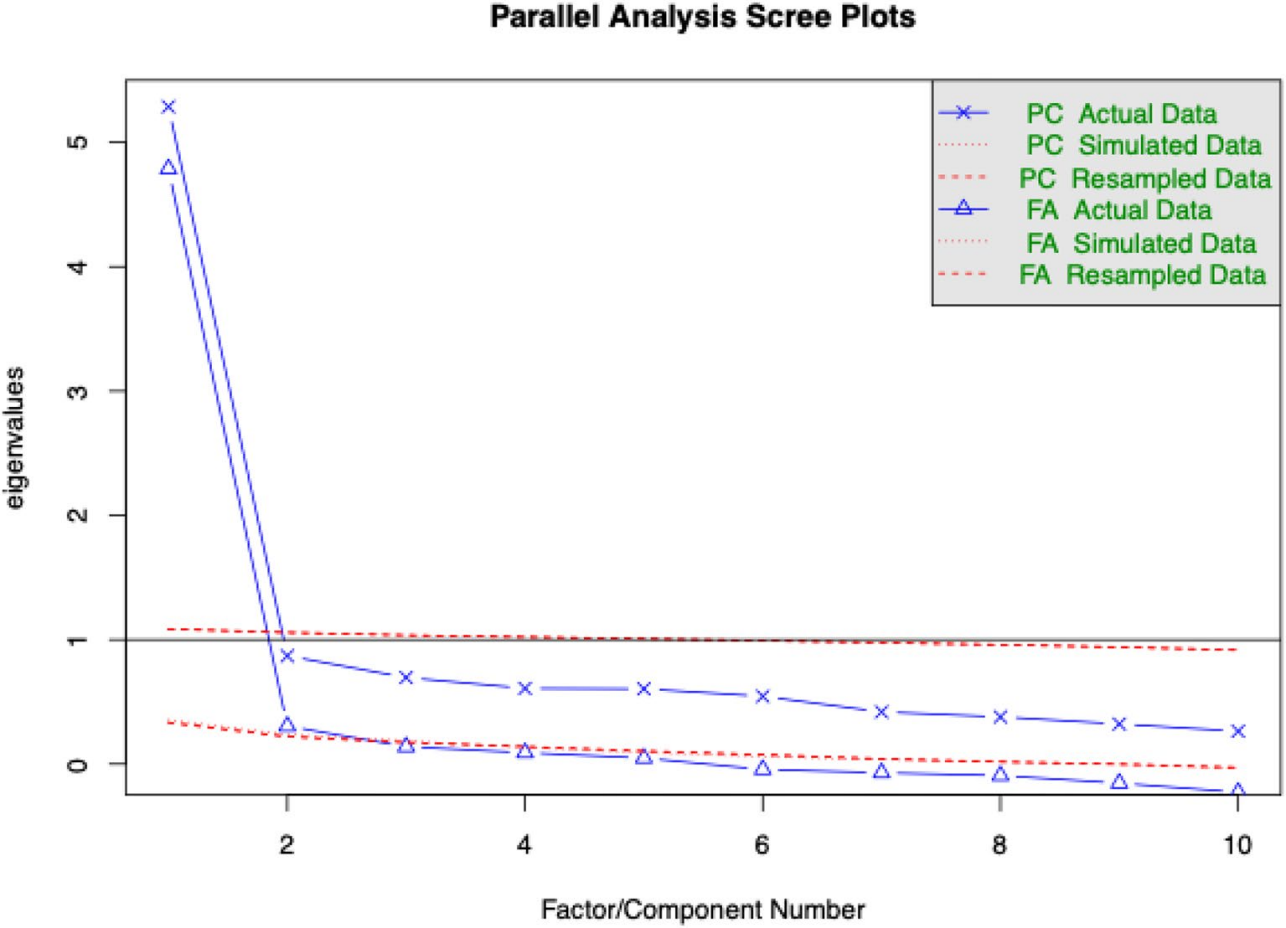
Parallel analysis scree and 2-factor correlation plots of the Spanish for Ecuador FROM-16

Parallel factor analysis showed a scree plot with a change in slope indicating two factors and two Eigenvalues were reported greater than 1 (Fig. 1).

The CFA of the 2-factor model of FROM-16 revealed a RMSEA of 0.071 (90% CI 0.057–0.085), SRMR value of 0.061, CFI of 0.90 and TLI 0.89 (Table 2), superior to a 1-factor model, indicating the same 2-factor structure as the original FROM-16. Very Simple structure (VSS) complexity 2 also achieved a maximum of 0.86 with 2 factors, [21, 22] and a minimum of 0.02 with 2 factors using Velicer’s Minimum Average Partial (MAP) Test [23].

**Table 2:**
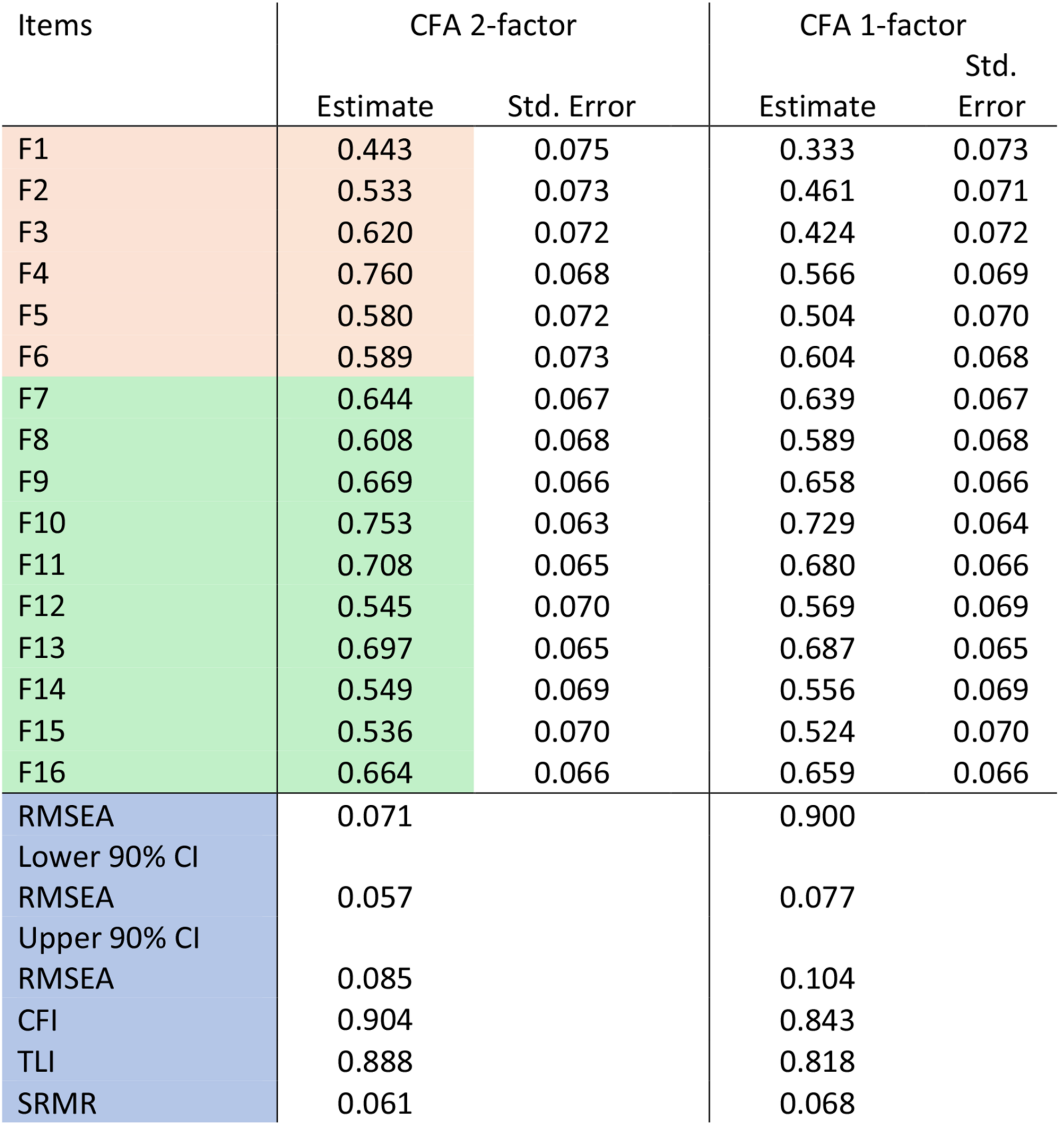
Confirmatory factor analysis of the Spanish for Ecuador FROM-16. Emotional state domain items are highlighted in pink, social and personal life domain items in green, and fit metrics in blue

#### Cronbach’s alpha-reliability

Overall Cronbach’s alpha was 0.890 for all 16 items, 0.758 for the emotional domain (items 1–6) and 0.873 for the personal and social life domain (items 11–16). Cronbach’s alpha did not increase if any single items were deleted.

#### Item response theory

**Fig. 2:**
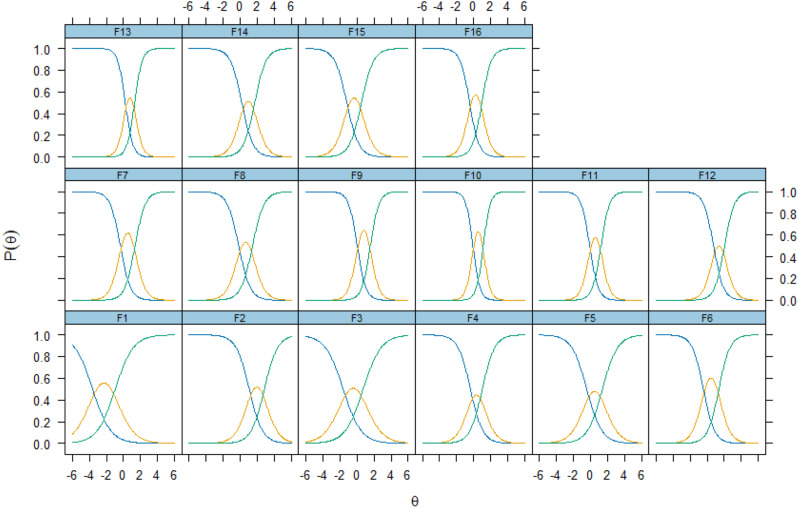
Item response curves for Spanish for Ecuador FROM-16

All items showed good fit in the IRT model with factor loadings between 0.443 and 0.760 (cut-off > 0.36) and S-X^2^ item level diagnostic statistics indicated optimal fit for items with *p* < 0.05 and S-X^2^ RMSEA < 0.05 for all items. Overall fit statistics were: RMSEA 0.09, SRMSR 0.077, TLI 0.93, and CFI 0.94. Item trace lines indicated no misfitting items and all items had infit/outfit t (-2 to + 2) in a Rasch model. Spanish for Ecuador FROM-16 shows no local dependencies (all LDG2 < 0.2 and almost all Q3 below the accepted cut-off of 0.37; item 3 and 4 correlation was 0.39) [24]. Items were well distributed along the ability scale (theta) from − 2.29 to 1.95 indicating the measure’s ability to capture a wide range of responses (Fig. 2).

Figure 2 demonstrates the item response curves for each of the FROM-16 questions i.e. the modelled probability of a correct answer to the item for respondents at each point in the ability range. The curves show excellent fit; for every item there is a location (ability) where each response level is the most likely response i.e., no curve is completely enveloped beneath others.

Additional data are given in the Supplement Data tables and figures including item score distributions, descriptive statistics, the Ecuador FROM-16 score distribution, Cronbach’s alpha analysis, Rasch model infit and outfit and fit statistics, Graded Response Model (GRM) parameters, item and model fit statistics, local dependence (LD) matrix, Q3 summary statistics and item anchor points on the theta (ability) scale.

### Part II - Clinical application of the Ecuadorian FROM-16

#### Study participants recruitment and response rate

The patients who were most often accompanied by FMs were those at the extremes of age (i.e. paediatric and elderly patients). A total of 200 FMs were recruited into the study. Five forms were discarded: one as the FM withdrew consent, three were returned unfilled, and one form was only partially completed. Three other FMs initially returned incomplete questionnaires, all with only one question answered per domain: on further explanation these were then fully completed. Of the 195 fully completed FROM-16 questionnaires, 187 were completed without assistance and eight FMs requested help filling out the questionnaire, six because of illiteracy (*n* = 6) and two because they stated they had not brought their reading glasses (*n* = 2). The mean time to complete the FROM-16, measured in 14 cases, was 136 s (SD = 39.6, range 74–200).

#### Demographic and clinical characteristics of study participants

Patients’ mean age was 41.8 ± 31years (range 0-100), 109 female (56%), and 86 male (44%). Skin health status was recorded using a five-point Likert scale by FMs and by a sole physician to remove bias due to inter-rater variability. The skin health status was rated better by the physician than by the FMs (Table 3). The interclass correlation coefficient was calculated in SPSS using a two-way mixed effect and single measures model giving an interclass correlation (ICC) of 0.493 (95% CI -0.379 to 0.592) *p* < 0.001. The intraclass correlation of 0.493 indicates only poor to fair agreement between raters. Using Cohen’s kappa as a measure of the agreement between raters gave κ = 0.155 (*p* < 0.001) indicating poor strength of agreement [25].

**Table 3:** Rating of the patients’ current skin health status by family members and physician

Skin health status	Ratings by family member (*n* = 195)	Ratings by physician (*n* = 1)
Very good	24	39
Good	58	81
Fair	70	63
Poor	31	8
Very poor	12	4

The mean age of FMs was 47 ± 7 years (median 45, range 18–88), greater than that of patients (41.8, *p* = 0.05). FMs comprised 149 women (76%), and 46 men (24%); 92 were parents of the person with skin disease; 74 were mothers and 18 fathers; 55 children, 34 spouses, 8 siblings, 5 grandmothers, 1 niece and 1 cousin. Of the 195 FMs, 144 (73.8%) considered themselves the main patient caregiver and 152 (77.9%) cohabited with the patient (Table 4).

**Table 4:** Demographic characteristics of the study participants

**Family members**	
Number	195
Mean age, years	47.3 ± 7
Median age, years	45
Age range, years	18–88
Females (n, %)	149 (76%)
Males	46 (24%)
**Relationship of FM to patient**	
Mother	74 (37.9%)
Adult children	54 (28.2%)
Spouses	34 (17.4%)
Father	18 (9.2%)
Other (sibling, grandmother, aunt, niece)	15 (7.7%)
FMs who lived in the same household	152 (77.9%)
FMs who considered themselves the main patient carer	144 (73.8%)
**Patients**	
Number	195
Mean age, years	41.8 ± 31.1
Median age, years	38
Age range, years	0-100
Females	109 (56%)
Males	86 (44%)
**Skin disease**	
Inflammatory	88
Papulo-squamous and eczematous disorders (psoriasis, lichen planus, pityriasis versicolor, atopic and contact dermatitis)	51
Adnexal disease (acne and alopecia areata)	17
Collagen vascular disease (lupus erythematosus, localized and systemic sclerosis, erythema nodosum)	9
Pigmentary disorders (vitiligo)	3
Geno dermatoses (xeroderma pigmentosa, epidermolysis bullosa)	3
Cutaneous lymphoma	3
Bullous skin diseases (bullous pemphigoid and pemphigus vulgaris)	2
Non-Inflammatory	107
Skin cancer and precursors (46 squamous cell carcinoma and basal cell carcinoma, 8 melanoma, 5 actinic keratosis, 1 Kaposi sarcoma, 1 extramammary Paget´s)	61
Infectious disease (viral warts, molluscum contagiosum, onychomycosis, impetigo, herpes zoster)	14
Pigmentary skin disorders (café au lait macules, nevi, melanonychia, pseudo acanthosis nigricans)	12
Benign skin tumour (2 pilomatricomas, 2 seborrheic keratoses, 1 verrucous epidermal nevus, 1 nevus sebaceous, 1 infantile hemangioma, 1 mucocele, 1 nail tumour)	9
Xerosis, pityriasis alba and keratosis pilaris	3
Adnexal disease (hyperhidrosis)	2
Genodermatosis (tuberous sclerosis)	1
Unknown diagnosis	5

#### FROM-16 scores

**Fig. 3:**
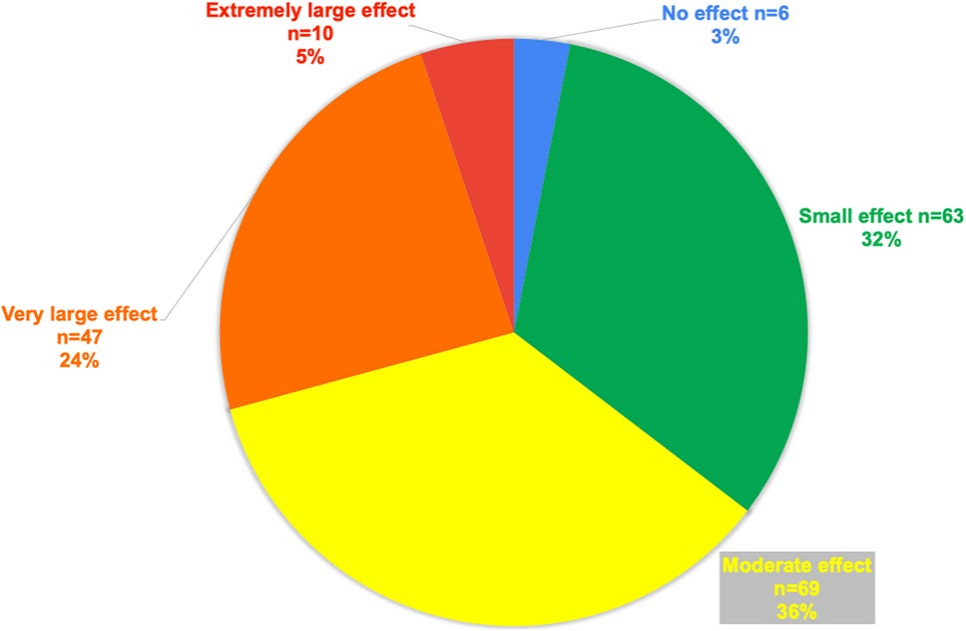
Percentage of FMs experiencing each level of impact on QoL

The score band descriptor [18] aligned to the greatest number of FMs of patients indicated a moderate effect on their QoL (Fig. 3), mean FROM-16 = 12.5 (SD ± 7, median 12, range 0–31, *n* = 195), and the impact was similar between females (mean FROM-16 = 12.6, SD ± 7.3; *n* = 149) and males (mean FROM-16 = 12.4, SD ± 6.1; median 11, *n* = 46) (*p* = 0.99). Similarly, there were no significant differences in impact on QoL between main caregivers (12.8 ± 6.9, *n* = 144, 73.8%) and FMs who were not main caregivers (12 ± 7.3, *n* = 51, 28.2%, *p* = 0.47). Family members recorded increased worry, sadness and frustration in the emotional domain; and, in the personal and social domain, the greatest concerns regarded family expenses, sleep and “finding time for oneself.”

FMs not cohabiting were not significantly more affected (mean FROM-16 13.1 ± 7.6, 43 of 195) than cohabitants (mean 12.5 ± 6.9, 152 of 195) (*p* = 0.71). However, FMs of patients with inflammatory conditions had a higher mean score (14 ± 6.9, *n* = 88, 45.1%) than FMs of those with non-inflammatory conditions (11.4 ± 7, *n* = 107, 54.8%, *p* = 0.006).

Adult children of patients (*n* = 54, mean FROM-16 score 13.4 ± 7.4), were more affected than parents (*n* = 92, mean = 12.6 ± 6.9, *p* > 0.05). Adult children who were main caregivers not cohabiting with the parent experienced a particularly high impact on QoL (mean FROM-16 = 17 ± 7.7, *n* = 8) compared to those who cohabited (11.6 ± 6.7, *n* = 11, *p* = 0.05). When the patients’ health was described as “poor” or “very poor” by FMs the mean FROM-16 score = 16.6 (*n* = 46). If the patients’ health was described as “poor” or “very poor” by the physician the mean FROM-16 score = 19.7 (*n* = 12). However, when FMs described the patient´s skin status as “Good” or “Very Good” the mean FROM-16 score was 10.7 (*n* = 82, *p* < 0.05): similarly, when the physician described the patient´s skin status as “Good” or “Very Good” the mean FROM-16 score was 10.7 (*n* = 82, *p* < 0.05) (see Table 5).

**Table 5:** Comparisons of quality of life impact on different family relationships

Family member group	Number of FMs in each group	Mean FROM-16 score	*p* value
Adult children of patients (main carers and not main carers)	54	13.4	
Parents of patients	92	12.6	*p* > 0.05
Main carer adult children cohabiting with (patient) parent	11	11.6	*p* = 0.05
Main carer adult children not cohabiting with (patient) parent	8	17.0	
FMs of patients with inflammatory conditions	88	14.0	
FMs of patients with non-inflammatory conditions	107	11.4	*p* = 0.006
FM rating skin health status as good or very good	82	10.7	
FM rating skin health status as poor or very poor	46	16.6	*p* < 0.05
Physician rating skin health status as good or very good	120	10.9	
Physician rating skin health status as poor or very poor	12	19.7	*p* < 0.05
Male FMs	46	12.4	
Female FMs	149	12.6	*p* = 0.99
FM, main carer	144	12.8	
FM, not main carer	51	12.0	*p* = 0.47

Family members aged between 18 and 59 years, had a mean FROM-16 score of 13 ± 6.7 (*n* = 153), and FMs aged 60 and above had a mean FROM-16 score of 11 ± 7.9 (*n* = 42) (*p* = 0.10). Family members had similar mean FROM scores (range 12.0 to 14.5) across different patient age spans (Table 6), except for the two FMs of infants (mean = 2.5).

**Table 6:** Mean FROM-16 score and interpretation of the degree of impact on different family member groups, using validated score meaning bands [18]

Patient group	Number	FROM-16 score (0–32)	Mean emotional score (0–12)	Mean personal and social score (0–20)	Score meaning: effect on quality of life
All patients	195	12.5 ± 7	5.5 ± 2.8	7.1 ± 5	moderate effect
Females	149	12.6 ± 7.3	5.5 ± 2.9	7.1 ± 5.1	moderate effect
Males	46	12.4 ± 6.1	5.3 ± 2.8	7.0 ± 4.9	moderate effect
Adult children	54	13.4 ± 7.4	5.7 ± 2.7	7.7 ± 5.2	moderate effect
Parents	92	12.6 ± 6.9	5.5 ± 2.8	7.1 ± 4.8	moderate effect
Mothers	74	13.2 ± 7.2	5.7 ± 2.9	7.4 ± 4.9	moderate effect
Fathers	18	10.4 ± 4.8	4.6 ± 1.9	5.8 ± 3.8	moderate effect
FM cohabiting	152	12.5 ± 6.9	5.5 ± 2.8	7 ± 4.8	moderate effect
FM not cohabiting	43	13.1 ± 7.6,	5.8 ± 2.9	7.3 ± 5.4	moderate effect
Main caregivers	144	12.8 ± 6.9	5.6 ± 2.8	7.2 ± 4.9	moderate effect
Not main caregivers	51	12 ± 7.3	5.3 ± 2.9	6.7 ± 5	moderate effect
Adult children, main caregivers and cohabiting	11	11.63 ± 6.7	5.9 ± 2.3	5.7 ± 4.4	moderate effect
Adult children, main caregivers not cohabiting	8	17 ± 7.7	6.1 ± 2	10.9 ± 4	very large effect
Adult children, not main caregivers and cohabiting	11	15.5 8.4	5.9 ± 3.1	9.6 ± 5.6	moderate effect
Adult children, not main caregivers, not cohabiting	24	12.1 7.4	5.4 ± 2.9	6.7 ± 5.1	moderate effect
FMs of patients with inflammatory conditions	88	14 ± 6.9	6.0 ± 3	7.9 ± 4.6	moderate effect
FM of those with non-inflammatory conditions	107	11.4 ± 7	5.1 ± 2.6	6.3 ± 5.1	moderate effect
FM age18- 59 years	153	13 ± 6.7	5.7 ± 2.7	7.4 ± 4.8	moderate effect
FM aged 60 or over	42	11 ± 7.9	5.1 ± 3.3	6 ± 5.4	moderate effect
FM of infants age less than 1	2	2.5 ± 1.5	1.5 ± 0.5	1 ± 1	small effect
FM of children ages 1 to 9	33	12 ± 6.1	5.4 ± 2.4	6,6 ± 4.6	moderate effect
FM of those aged 10–19	52	12.7 ± 6.4	5.5 ± 2.6	7.2 ± 4.4	moderate effect
FM of adults (20–59),	25	14.5 ± 7.5	6 ± 3.3	8.4 ± 5	moderate effect
FM of those aged 60–79,	55	13 ± 7.6	5.8 ± 3	7.2 ± 5.4	moderate effect
FM of those aged 80 and above,	28	11.6 ± 7	5.2 ± 2.6	6.4 ± 5	moderate effect

#### FROM-16 score banding

**Fig. 4:**
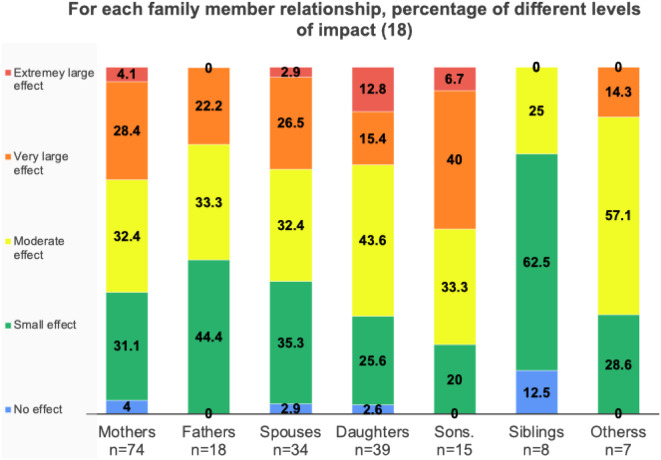
FROM-16 score banding among different FMs

Only 4% of FMs experienced no effect on their QoL (7 of 195), 32% (*n* = 63) experienced a “small effect”, and 36% (*n* = 69) a “moderate effect”. However, 29.2% of FMs (*n* = 57) experienced a “very large” (*n* = 47) or “extremely large” (*n* = 10) effect on QoL. 65% of mothers (48 out of 74) and 55% of fathers (*n* = 10, out of 18, *p* = 0.65) experienced a moderate to extremely large effect on their QoL. 80% of sons, 72% of daughters, 71% of other siblings and 62% of spouses had a moderate to extremely large effect on their QoL. Siblings reported the lowest impact, with only 25% reporting a moderate to extremely large impact (Table 7; Fig. 4).

**Table 7:**
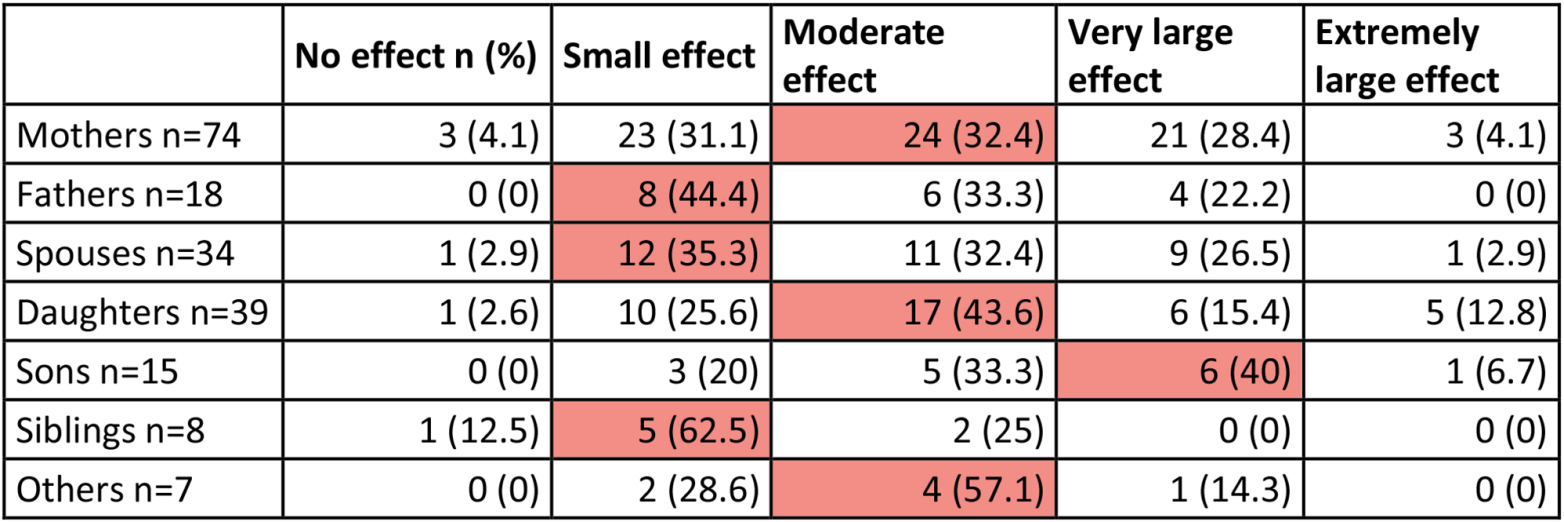
Score banding in different family member categories. Red is used to highlight the most frequently reported level of effect within each family member category

## Discussion

### Psychometric analysis

CFA provided evidence to support the suggested 2-factor structure for the Spanish for Ecuador FROM-16. The RMSEA (0.071) was within the acceptable levels of error of approximation (> 0.05–0.08 = good fit), while the SRMR value (0.061) was below the accepted cut-off < 0.08 indicating good fit. The CLI fit statistic (0.90) indicated acceptable fit (0.90–0.95) whilst TLI (0.888) was slightly below desired cut-off. The sample size for this study was smaller than desired for the analyses performed; however, the number of subjects analysed (*n* = 195) was well above the recommendation according to COSMIN, of the minimum requirement of a sample size to conduct CFA as 7 subjects per item (7 × 16 items = 112 subjects) [26, 27]. Internal consistency as calculated by Cronbach’s alpha of 0.89 confirms the internal consistency (reliability) of the measure [28]. IRT showed excellent fit to Rasch and graded response models, with no misfitting items or local dependence. The Spanish for Ecuador FROM-16 has been shown to have strong structural validity. These findings, along with the validated score meaning banding [18], the validated minimal important score difference of four points [29] and the ability to map FROM-16 scores to EQ-5D scores for utility calculations [30], confirm the usefulness of FROM-16.

### Limitations

Ideally, after a validated translation of a questionnaire is carried out, a final process of cultural adaptation or of confirming the cultural validity of a questionnaire should be carried out amongst a series of subjects, representative of the population in which the questionnaire is to be used. In this study a detailed process of cognitive debriefing was carried out on 54 FMs, using a separate structured questionnaire: the results of this provide strong reassurance that the questionnaire is appropriate for use in this population. The addition of a focus group may have made this process even stronger.

### Clinical application

Patients with diseases across every medical specialty can affect the emotional, financial and social well-being of family members [1, 31–34]. However, dermatology patients can have an extra impact on the time the FM spends in caring, for example helping with frequent application of topical therapy [7–12, 35, 36]. Moreover, care for linen and clothing of a dermatology patient may be more demanding than for other household members. Frequent visits to dermatology clinics may result in the FM changing their daily activities or being absent from work [36–38]. However, FMs may become worried, sad and frustrated even if they are not main caregivers, and carry a high burden concerning family expenses, interrupted sleep and finding time for themselves, as demonstrated by our study and others [39, 40].

This study reinforces the importance of QoL studies in FMs as although there is, on average, a “moderate impact” upon FMs when someone has a skin condition, this generalisation masks the large number of individual FMs experiencing a very large impact on their QoL. Inflammatory skin diseases can affect FMs more than a relative having skin cancer (Table 8) [39]. Our study revealed that all FMs carry a burden in their QoL, and the most affected are FMs of patients with inflammatory skin disease, those whose health is rated as poor or very poor by physicians or FMs, and adult children who do not live in the same house as their sick parents. Furthermore, some FMs are very affected even if the patients’ skin health is rated good or very good.

Importantly, the results highlight a consistent discrepancy between physician and FM assessments of skin health. Physicians often rate the condition as milder (ICC = 0.493, κ = 0.155, both *p* < 0.001), likely due to their clinical detachment and reliance on objective criteria. In contrast, FMs, who are more attuned to the patient’s emotional and day-to-day struggles, may offer a more comprehensive and accurate assessment of disease severity. This stresses the importance of their perspective in treatment decisions.

Our FROM-16 scores are compared to those of other studies (Table 8). Our study in dermatology reported similar FROM-16 scores to a study of FMs of patients with general diseases [16]. Moreover, the FROM-16 demonstrates a higher impact in chronic general medical conditions than in skin conditions [18, 40, 41]. Our study recorded similar scores (mean FROM-16 score = 12.6) to the Thai study of FMs of 248 cancer patients (mean = 11.8) [39]. 

**Table 8:** Comparison between different FROM-16 studies

Study	Subject characteristics	Subject Number	MeanFROM-16 score,(range0–32)	Median	Range	Emotional domain (range 0–12)	Personal and social life Domain (range 0–20)
Chantarasap et al [39]	FMs of cancer patients	248	11.8 ± 5.9	11	1–31	4.7 ± 2.5	7.1 ± 3.4
Elsner et al [41]*	FMs of patients with chronic disease	83	16.8 ± 6.6	17	0–31		
Shah [18]	FMs of patients with diverse health conditions	4413	15 ± 8.1		0–32		
Shah [42]	FMs of COVID-19 survivors	735	15	15	0–32	6.1	8.9
Golics et al [16]	FMs of patients with diseases from 26 specialties	240	12.3 ± 7.5	11.5	1–31	5.6	6.7
Vyas [40]	FMs of patients with myalgic encephalomyelitis/ chronic fatigue syndrome	1418	17.9 ± 7	18		7.6 ± 2.8	10.3 ± 4.9
Our Study	FMs of patients with any skin condition	195	12.6 ± 7	12	0–31	5.5 ± 2.8	7.1 ± 5

*Elsner et al., first analysis at t1. Data missing was not reported in the cited studies

Adult children were more impacted than parents, and those adult children who were main caregivers who do not live in the same household (mean FROM-16 score = 17) experienced a greater burden than those who cohabited with the patient (mean 11.6). Family members of patients aged 20–59 years faced a high burden (mean = 14.5), possibly as this is a working-age group and patients may not be as productive as expected, or families may be spending more on treatment than on family activities.

In our study the mean FROM-16 completion time was 2 min 16 s, concordant with the original FROM-16 study [16, 17] and re-affirming that FROM-16 is an easy-to-use tool which could, for example, be completed while a family member and patient are in the waiting room. Moreover, FMs are overall willing to complete the FROM-16 and being asked to do this by the healthcare provider may make them feel more involved.

Some FMs reported feelings of guilt, believing that they should not feel anger or frustration regarding the patient’s condition. Perhaps it is “assumed” by society that family members should care for those unwell in their family, without any negative feelings.

Interventions to address this family impact cannot solely be the physician´s responsibility; signposting to educational programmes [43, 44] and mindfulness programmes [45], as a complement to consultations are possible approaches.

Not only does the FROM-16 emphasise the impact on FMs when someone is ill, but health economists recommend including FMs in economic evaluations. A study performed by Shah et al. [30] demonstrates that the FROM-16 scores can be used to calculate Euroqol EQ-5D utility values for economic appraisals. The outcome of such exercise could then guide policy makers as well payers with their economic and reimbursement decisions regarding healthcare provisions.

## Conclusions

This study reveals the considerable burden experienced by FMs of dermatology patients, regardless of the specific diagnosis. These results challenge dermatologists to develop effective ways to alleviate this “secondary” QoL impact. FROM-16 could be used to identify those FMs who need extra support. This may be very important to enhance the quality of care of patients, as FMs are often key to concordance with therapy.

As healthcare providers, it is essential to recognize where our scope of care should expand to better support FMs. Tools with strong validity and internal consistency, such as the FROM-16, are invaluable for accurately portraying the broader impact of disease. As the physician Matt McCarthy said: ¨Families, in some ways, became our second set of patients. They needed time and attention, and if you failed to provide that, things could deteriorate quickly [46].

## Electronic supplementary material

Below is the link to the electronic supplementary material.

Supplementary Material 1

Supplementary Material 2

## Data Availability

Data is available upon reasonable request.

## Abbreviations

FROM-16: Family Reported Outcome Measure
FMs: Family members and partners
QoL: Quality of life
M: Mean
SD: Standard deviation
Md: Median
CFI: Comparative fit index
TLI: Tucker–Lewis’s index
RMSEA: Root Mean Square Error of Approximation
SRMR: Standardised Root Mean Square Residual
CI: Confidence intervals
