# Supplementary material for: Psychometric validation of the Spanish for Ecuador Family Reported Outcome Measure (FROM-16) and its application to measure impact on family members of patients with skin diseases
Source: J Patient Rep Outcomes. 2025 May 9;9:51. doi: 10.1186/s41687-025-00866-5 (PMC12064521; doi:10.1186/s41687-025-00866-5)

Supplementary Tables and Figures

Table S1. Ecuador FROM-16 item score distributions.

|  | F1 | F2 | F3 | F4 | F5 | F6 | F7 | F8 | F9 | F10 | F11 | F12 | F13 | F14 | F15 | F16 |
| --- | --- | --- | --- | --- | --- | --- | --- | --- | --- | --- | --- | --- | --- | --- | --- | --- |
| Mean | 1.64 | 0.30 | 1.14 | 0.86 | 0.81 | 0.79 | 0.77 | 0.71 | 0.61 | 0.71 | 0.70 | 0.37 | 0.58 | 0.59 | 1.13 | 0.88 |
| Median | 2.00 | 0.00 | 1.00 | 1.00 | 1.00 | 1.00 | 1.00 | 1.00 | 0.00 | 1.00 | 1.00 | 0.00 | 0.00 | 0.00 | 1.00 | 1.00 |
| Std. Deviation | 0.571 | 0.552 | 0.744 | 0.806 | 0.767 | 0.724 | 0.725 | 0.740 | 0.690 | 0.754 | 0.750 | 0.625 | 0.738 | 0.715 | 0.755 | 0.774 |
| Skewness | -1.307 | 1.668 | -0.229 | 0.267 | 0.338 | 0.333 | 0.373 | 0.528 | 0.690 | 0.542 | 0.561 | 1.449 | 0.852 | 0.791 | -0.226 | 0.207 |
| Std. Error of Skewness | 0.174 | 0.174 | 0.174 | 0.174 | 0.174 | 0.174 | 0.174 | 0.174 | 0.174 | 0.174 | 0.174 | 0.174 | 0.174 | 0.174 | 0.174 | 0.174 |
| Kurtosis | 0.739 | 1.851 | -1.158 | -1.411 | -1.225 | -1.040 | -1.031 | -1.010 | -0.674 | -1.055 | -1.024 | 0.938 | -0.669 | -0.658 | -1.211 | -1.301 |
| Std. Error of Kurtosis | 0.346 | 0.346 | 0.346 | 0.346 | 0.346 | 0.346 | 0.346 | 0.346 | 0.346 | 0.346 | 0.346 | 0.346 | 0.346 | 0.346 | 0.346 | 0.346 |
| Minimum | 0 | 0 | 0 | 0 | 0 | 0 | 0 | 0 | 0 | 0 | 0 | 0 | 0 | 0 | 0 | 0 |
| Maximum | 2 | 2 | 2 | 2 | 2 | 2 | 2 | 2 | 2 | 2 | 2 | 2 | 2 | 2 | 2 | 2 |

Table S2. Ecuador FROM-16 descriptive statistics

|  | N | Range | Mean | Std. Deviation | Median | Floor (%) | Ceiling (%) |
| --- | --- | --- | --- | --- | --- | --- | --- |
| Total score (0-30) | 195 | 0-32 | 12.59 | 7.050 | 12 | 1 | 0.5 |
| Emotional domain | 195 | 0-12 | 5.54 | 2.829 | 5 | 2.1 | 2.6 |
| Personal and Social domain | 195 | 0-20 | 7.06 | 4.968 | 6 | 7.7 | 1.5 |

Figure S1. Ecuador FROM-16 score distribution

‘
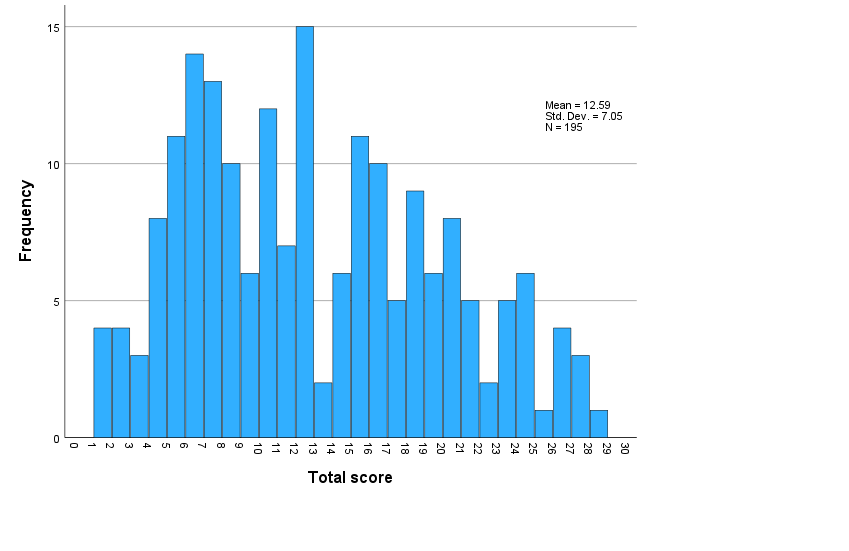


Table S3. Cronbach’s alpha

|  | Scale Mean if Item Deleted | Scale Variance if Item Deleted | Corrected Item-Total Correlation | Squared Multiple Correlation | Cronbach's Alpha if Item Deleted |
| --- | --- | --- | --- | --- | --- |
| F1 | 10.96 | 46.720 | 0.341 | 0.241 | 0.890 |
| F2 | 12.29 | 46.022 | 0.451 | 0.277 | 0.887 |
| F3 | 11.46 | 44.744 | 0.443 | 0.459 | 0.887 |
| F4 | 11.74 | 42.967 | 0.576 | 0.486 | 0.882 |
| F5 | 11.78 | 44.056 | 0.497 | 0.389 | 0.885 |
| F6 | 11.80 | 43.645 | 0.578 | 0.362 | 0.882 |
| F7 | 11.82 | 43.633 | 0.579 | 0.505 | 0.882 |
| F8 | 11.89 | 43.833 | 0.543 | 0.433 | 0.883 |
| F9 | 11.98 | 43.757 | 0.599 | 0.448 | 0.881 |
| F10 | 11.89 | 42.637 | 0.660 | 0.571 | 0.879 |
| F11 | 11.90 | 43.031 | 0.621 | 0.536 | 0.880 |
| F12 | 12.22 | 44.791 | 0.541 | 0.358 | 0.884 |
| F13 | 12.02 | 42.953 | 0.642 | 0.470 | 0.879 |
| F14 | 12.01 | 44.253 | 0.520 | 0.341 | 0.884 |
| F15 | 11.46 | 44.270 | 0.484 | 0.306 | 0.886 |
| F16 | 11.71 | 42.814 | 0.621 | 0.452 | 0.880 |

Table S4. Infit and outfit and fit statistics (Rasch model). All items have infit/outfit t(-2 to +2)

| item | outfit | | z.outfit | | infit | | z.infit | |  |  |  |  |  |
| --- | --- | --- | --- | --- | --- | --- | --- | --- | --- | --- | --- | --- | --- |
| F1 | 1.332 | | 1.838 | | 1.104 | | 0.929 | |  |  |  |  |  |
| F2 | 0.916 | | -0.335 | | 1.001 | | 0.051 | |  |  |  |  |  |
| F3 | 1.083 | | 0.875 | | 1.091 | | 1.031 | |  |  |  |  |  |
| F4 | 0.862 | | -1.275 | | 0.931 | | -0.756 | |  |  |  |  |  |
| F5 | 1.023 | | 0.254 | | 1.046 | | 0.540 | |  |  |  |  |  |
| F6 | 0.866 | | -1.415 | | 0.895 | | -1.194 | |  |  |  |  |  |
| F7 | 0.881 | | -1.221 | | 0.892 | | -1.219 | |  |  |  |  |  |
| F8 | 0.907 | | -0.824 | | 0.960 | | -0.413 | |  |  |  |  |  |
| F9 | 0.799 | | -1.828 | | 0.858 | | -1.534 | |  |  |  |  |  |
| F10 | 0.711 | | -2.755 | | 0.781 | | -2.533 | |  |  |  |  |  |
| F11 | 0.794 | | -1.874 | | 0.834 | | -1.866 | |  |  |  |  |  |
| F12 | 0.807 | | -0.976 | | 0.886 | | -0.954 | |  |  |  |  |  |
| F13 | 0.677 | | -2.476 | | 0.801 | | -2.119 | |  |  |  |  |  |
| F14 | 0.897 | | -0.775 | | 1.007 | | 0.102 | |  |  |  |  |  |
| F15 | 1.039 | | 0.428 | | 1.024 | | 0.301 | |  |  |  |  |  |
| F16 | 0.788 | | -2.257 | | 0.835 | | -1.960 | |  |  |  |  |  |
| M2 | | df | | p | | RMSEA | | RMSEA_5 | | RMSEA_95 | SRMSR | TLI | CFI |
| 303.7656 | | 103 | | <0.001 | | 0.100 | | 0.087 | | 0.113 | 0.093 | 0.910 | 0.911 |

Table S5. Graded Response Model (GRM) parameters, item and model fit statistics.

|  | a | b1 | b2 | | d1 | d2 | | S_X^2^ df | | df.S_X^2^ | | RMSEA.S_X^2^ | | p.S_X^2^ | | X^2^ d | | df.X^2^ | RMSEA.X^2^ | | p.X^2^ | | PV_Q1 df. |
| --- | --- | --- | --- | --- | --- | --- | --- | --- | --- | --- | --- | --- | --- | --- | --- | --- | --- | --- | --- | --- | --- | --- | --- |
| F1 | 0.948 | -3.609 | -0.962 | | 3.421 | 0.912 | | 10.17 | | 14 | | 0.000 | | 0.750 | | 20.74 | | 17 | 0.034 | | 0.238 | | 23.62 |
| F2 | 1.292 | 1.064 | 2.84 | | -1.375 | -3.669 | | 22.63 | | 15 | | 0.050 | | 0.092 | | 5.97 | | 17 | 0.000 | | 0.993 | | 14.13 |
| F3 | 0.956 | -1.608 | 0.734 | | 1.538 | -0.702 | | 29.47 | | 29 | | 0.009 | | 0.441 | | 7.42 | | 17 | 0.000 | | 0.977 | | 15.80 |
| F4 | 1.377 | -0.374 | 1.02 | | 0.515 | -1.405 | | 33.70 | | 24 | | 0.046 | | 0.090 | | 16.16 | | 17 | 0.000 | | 0.513 | | 15.33 |
| F5 | 1.136 | -0.419 | 1.417 | | 0.476 | -1.609 | | 27.51 | | 28 | | 0.000 | | 0.491 | | 6.68 | | 17 | 0.000 | | 0.987 | | 16.23 |
| F6 | 1.536 | -0.434 | 1.379 | | 0.667 | -2.119 | | 30.54 | | 22 | | 0.045 | | 0.106 | | 15.18 | | 17 | 0.000 | | 0.582 | | 17.55 |
| F7 | 1.683 | -0.36 | 1.354 | | 0.607 | -2.278 | | 28.62 | | 22 | | 0.039 | | 0.156 | | 16.86 | | 17 | 0.000 | | 0.464 | | 15.50 |
| F8 | 1.481 | -0.165 | 1.449 | | 0.245 | -2.146 | | 20.67 | | 24 | | 0.000 | | 0.658 | | 18.81 | | 17 | 0.023 | | 0.339 | | 18.16 |
| F9 | 1.984 | 0.038 | 1.574 | | -0.075 | -3.122 | | 14.27 | | 20 | | 0.000 | | 0.817 | | 18.10 | | 17 | 0.018 | | 0.383 | | 19.09 |
| F10 | 2.457 | -0.074 | 1.129 | | 0.183 | -2.773 | | 23.03 | | 19 | | 0.033 | | 0.236 | | 17.51 | | 17 | 0.012 | | 0.421 | | 18.96 |
| F11 | 1.997 | -0.079 | 1.24 | | 0.158 | -2.476 | | 18.27 | | 20 | | 0.000 | | 0.570 | | 13.99 | | 17 | 0.000 | | 0.668 | | 16.09 |
| F12 | 1.724 | 0.758 | 2.039 | | -1.308 | -3.515 | | 8.82 | | 17 | | 0.000 | | 0.946 | | 8.57 | | 17 | 0.000 | | 0.953 | | 15.38 |
| F13 | 2.258 | 0.219 | 1.305 | | -0.493 | -2.946 | | 25.19 | | 20 | | 0.037 | | 0.194 | | 10.40 | | 17 | 0.000 | | 0.886 | | 18.16 |
| F14 | 1.51 | 0.197 | 1.704 | | -0.297 | -2.572 | | 33.54 | | 23 | | 0.049 | | 0.072 | | 31.10 | | 17 | 0.065 | | 0.019 | | 22.49 |
| F15 | 1.42 | -1.184 | 0.534 | | 1.681 | -0.758 | | 25.39 | | 25 | | 0.009 | | 0.441 | | 28.13 | | 17 | 0.058 | | 0.043 | | 29.99 |
| F16 | 1.815 | -0.477 | 0.96 | | 0.865 | -1.743 | | 20.15 | | 22 | | 0.000 | | 0.574 | | 21.00 | | 17 | 0.035 | | 0.226 | | 16.00 |
| M2 | | | | df | | | p | | RMSEA | | RMSEA_5 | | RMSEA_95 | | SRMSR | | TLI | | | CFI | |  |  |
| 226.64 | | | | 88 | | | <0.001 | | 0.090 | | 0.076 | | 0.104 | | 0.077 | | 0.927 | | | 0.938 | |  |  |

| 'S_X^2^': Orlando and Thissen (2000, 2003) and Kang and Chen's (2007) signed chi-squared test |
| --- |
| 'X^2^': Bock's (1972) chi-squared method. The default inputs compute Yen's (1981) Q1 variant of the X^2^ statistic (i.e., fixed group.bins = 10). |
| 'PV_Q1': Chalmers and Ng's (2017) plausible-value variant of the Q1 statistic. |
| 'X^2^*' : Stone's (2000) fit statistics that require parametric bootstrapping  df = degrees of freedom |

Table S6. Local dependence (LD) matrix (lower triangle) and standardized values (GRM).

|  | F1 | F2 | F3 | F4 | F5 | F6 | F7 | F8 | F9 | F10 | F11 | F12 | F13 | F14 | F15 | F16 |
| --- | --- | --- | --- | --- | --- | --- | --- | --- | --- | --- | --- | --- | --- | --- | --- | --- |
| F1 |  | 0.185 | 0.273 | 0.170 | -0.101 | 0.139 | -0.152 | -0.104 | -0.113 | -0.122 | -0.065 | 0.139 | 0.118 | -0.049 | -0.175 | 0.129 |
| F2 | 13.377 |  | 0.131 | 0.198 | 0.173 | -0.139 | 0.098 | -0.116 | 0.053 | -0.101 | -0.122 | -0.127 | -0.145 | -0.115 | -0.106 | -0.082 |
| F3 | 29.095 | 6.724 |  | 0.264 | 0.100 | 0.072 | -0.166 | -0.046 | -0.112 | -0.100 | -0.096 | 0.101 | -0.049 | 0.062 | -0.128 | -0.089 |
| F4 | 11.249 | 15.224 | 27.226 |  | 0.156 | 0.116 | -0.069 | -0.069 | -0.082 | -0.071 | -0.118 | 0.053 | -0.119 | 0.028 | -0.129 | 0.029 |
| F5 | 3.973 | 11.633 | 3.916 | 9.454 |  | 0.111 | 0.130 | -0.125 | 0.046 | -0.142 | -0.142 | 0.120 | -0.089 | -0.061 | -0.101 | 0.087 |
| F6 | 7.487 | 7.507 | 2.018 | 5.288 | 4.847 |  | 0.083 | -0.095 | -0.090 | -0.070 | -0.038 | 0.073 | -0.114 | 0.093 | -0.045 | -0.109 |
| F7 | 9.046 | 3.755 | 10.74 | 1.844 | 6.610 | 2.659 |  | 0.132 | 0.142 | 0.076 | 0.083 | -0.042 | -0.057 | 0.133 | -0.150 | 0.068 |
| F8 | 4.183 | 5.255 | 0.809 | 1.884 | 6.046 | 3.506 | 6.785 |  | 0.079 | -0.123 | 0.167 | -0.108 | 0.102 | -0.075 | 0.117 | -0.110 |
| F9 | 4.954 | 1.095 | 4.877 | 2.621 | 0.839 | 3.172 | 7.898 | 2.413 |  | 0.107 | -0.083 | -0.093 | -0.032 | -0.195 | -0.149 | 0.084 |
| F10 | 5.845 | 3.961 | 3.913 | 1.959 | 7.820 | 1.890 | 2.235 | 5.882 | 4.463 |  | 0.071 | 0.074 | 0.097 | 0.125 | -0.164 | -0.060 |
| F11 | 1.666 | 5.760 | 3.625 | 5.444 | 7.895 | 0.561 | 2.709 | 10.824 | 2.704 | 1.945 |  | -0.127 | 0.062 | -0.093 | 0.124 | 0.068 |
| F12 | 7.525 | 6.248 | 3.948 | 1.097 | 5.589 | 2.051 | 0.680 | 4.510 | 3.389 | 2.110 | 6.330 |  | -0.116 | -0.124 | -0.085 | 0.067 |
| F13 | 5.466 | 8.164 | 0.937 | 5.519 | 3.087 | 5.036 | 1.269 | 4.071 | 0.392 | 3.704 | 1.488 | 5.266 |  | 0.157 | 0.135 | -0.116 |
| F14 | 0.919 | 5.116 | 1.490 | 0.298 | 1.448 | 3.346 | 6.869 | 2.181 | 14.904 | 6.139 | 3.385 | 6.032 | 9.667 |  | -0.123 | -0.096 |
| F15 | 11.926 | 4.408 | 6.357 | 6.451 | 4.011 | 0.805 | 8.821 | 5.373 | 8.671 | 10.496 | 5.970 | 2.818 | 7.057 | 5.895 |  | 0.146 |
| F16 | 6.481 | 2.598 | 3.061 | 0.320 | 2.935 | 4.620 | 1.799 | 4.758 | 2.772 | 1.410 | 1.782 | 1.758 | 5.238 | 3.564 | 8.321 |  |

Table S7. Q3 summary statistics (GRM). Pink highlight >0.35

|  | F1 | F2 | F3 | F4 | F5 | F6 | F7 | F8 | F9 | F10 | F11 | F12 | F13 | F14 | F15 | F16 |
| --- | --- | --- | --- | --- | --- | --- | --- | --- | --- | --- | --- | --- | --- | --- | --- | --- |
| F1 |  | 0.075 | 0.331 | 0.142 | 0.007 | -0.010 | -0.260 | -0.066 | -0.120 | -0.125 | -0.090 | -0.032 | 0.041 | -0.052 | -0.088 | -0.022 |
| F2 | 0.075 |  | 0.115 | 0.195 | 0.037 | 0.006 | 0.050 | -0.103 | -0.027 | -0.094 | -0.159 | -0.001 | -0.225 | -0.124 | -0.103 | -0.142 |
| F3 | 0.331 | 0.115 |  | 0.393 | 0.132 | 0 | -0.366 | -0.050 | -0.200 | -0.239 | -0.112 | 0.034 | -0.032 | 0.038 | -0.100 | -0.055 |
| F4 | 0.142 | 0.195 | 0.393 |  | 0.214 | 0.055 | -0.112 | -0.164 | -0.127 | -0.195 | -0.286 | 0.013 | -0.195 | 0.004 | -0.173 | -0.066 |
| F5 | 0.007 | 0.037 | 0.132 | 0.214 |  | 0.044 | 0.119 | -0.141 | -0.053 | -0.320 | -0.247 | 0.114 | -0.049 | -0.069 | -0.107 | -0.028 |
| F6 | -0.010 | 0.006 | 0 | 0.055 | 0.044 |  | -0.046 | -0.129 | -0.123 | -0.110 | -0.107 | 0.078 | -0.137 | 0.032 | -0.081 | -0.152 |
| F7 | -0.260 | 0.050 | -0.366 | -0.112 | 0.119 | -0.046 |  | -0.066 | 0.001 | -0.035 | 0.061 | -0.132 | -0.176 | -0.047 | -0.132 | -0.054 |
| F8 | -0.066 | -0.103 | -0.050 | -0.164 | -0.141 | -0.129 | -0.066 |  | -0.031 | -0.157 | 0.236 | -0.228 | 0.050 | -0.092 | 0.112 | -0.188 |
| F9 | -0.120 | -0.027 | -0.200 | -0.127 | -0.053 | -0.123 | 0.001 | -0.031 |  | 0.110 | -0.113 | -0.008 | -0.147 | -0.199 | -0.086 | -0.082 |
| F10 | -0.125 | -0.094 | -0.239 | -0.195 | -0.320 | -0.110 | -0.035 | -0.157 | 0.110 |  | -0.077 | -0.046 | -0.017 | 0.066 | -0.115 | -0.158 |
| F11 | -0.090 | -0.159 | -0.112 | -0.286 | -0.247 | -0.107 | 0.061 | 0.236 | -0.113 | -0.077 |  | -0.226 | -0.073 | -0.154 | 0.020 | 0.045 |
| F12 | -0.032 | -0.001 | 0.034 | 0.013 | 0.114 | 0.078 | -0.132 | -0.228 | -0.008 | -0.046 | -0.226 |  | -0.158 | -0.073 | -0.030 | 0.050 |
| F13 | 0.041 | -0.225 | -0.032 | -0.195 | -0.049 | -0.137 | -0.176 | 0.050 | -0.147 | -0.017 | -0.073 | -0.158 |  | -0.018 | 0.020 | -0.150 |
| F14 | -0.052 | -0.124 | 0.038 | 0.004 | -0.069 | 0.032 | -0.047 | -0.092 | -0.199 | 0.066 | -0.154 | -0.073 | -0.018 |  | -0.144 | -0.086 |
| F15 | -0.088 | -0.103 | -0.100 | -0.173 | -0.107 | -0.081 | -0.132 | 0.112 | -0.086 | -0.115 | 0.020 | -0.030 | 0.020 | -0.144 |  | 0.075 |
| F16 | -0.022 | -0.142 | -0.055 | -0.066 | -0.028 | -0.152 | -0.054 | -0.188 | -0.082 | -0.158 | 0.045 | 0.050 | -0.150 | -0.086 | 0.075 |  |

Figure S2. Ecuador FROM-16 item anchor points on the theta (ability) scale


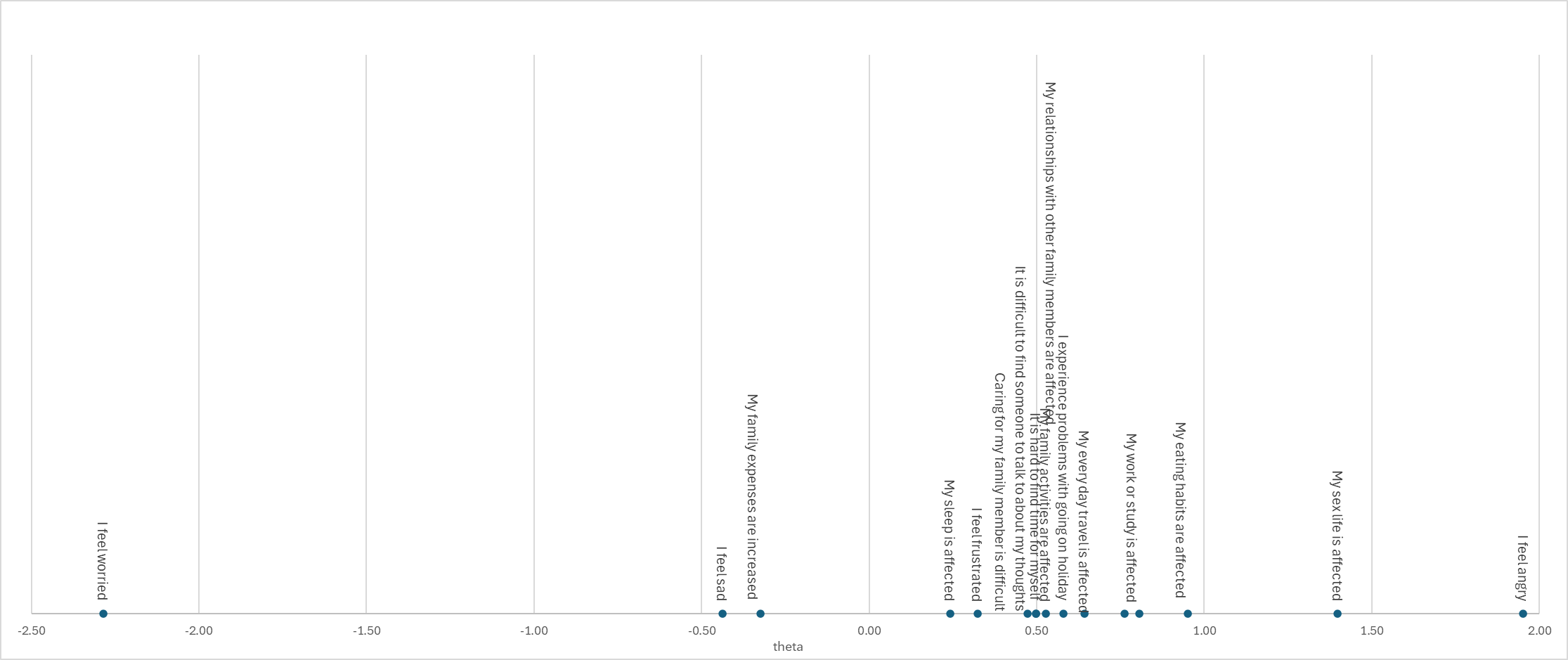

Supplement: Supplementary file 1 — Supplementary Material 1 [file 41687_2025_866_MOESM1_ESM.docx]
